# Early and Late Complications after Cataract Surgery in Patients with Uveitis

**DOI:** 10.3390/medicina59101877

**Published:** 2023-10-23

**Authors:** Gentian Bajraktari, Tomislav Jukić, Miro Kalauz, Martin Oroz, Andrea Radolović Bertetić, Nenad Vukojević

**Affiliations:** 1 Department of Ophthalmology, School of Medicine, University of Zagreb, 10000 Zagreb, Croatia; 2 Clinic of Ophthalmology, University Clinical Center of Kosovo, 10000 Prishtina, Kosovo; 3 Department of Ophthalmology, University Hospital Center Zagreb, 10000 Zagreb, Croatia

**Keywords:** uveitis-associated cataract, postoperative complications, phacoemulsification, uveitis, visual prognosis

## Abstract

*Background and Objectives*: Uveitis, a prevalent eye disorder characterized by inflammatory processes, often leads to cataract formation and significant visual impairment. This study aimed to evaluate preoperative conditions and postoperative outcomes following cataract surgery in uveitis patients. *Materials and Methods*: A retrospective study was conducted at the University Hospital Center Rebro Zagreb, Croatia, involving uveitis patients who underwent cataract surgery between 2013 and 2022. Eligible patients had uveitic cataracts affecting visual acuity or posterior segment visualization in a “quiet eye” and were disease-inactive for at least three months. Patients with certain pre-existing ocular conditions were excluded. The data collected included patient demographics, uveitis type, preoperative therapy, preexisting lesions, and postoperative outcomes such as visual acuity, intraocular pressure, central macular thickness, and complications. Statistical analysis was performed to identify risk factors associated with complications. *Results:* This study included 105 patients. The most common uveitis types were idiopathic uveitis, HLA-B27-associated uveitis, and JIA uveitis. After cataract surgery, there was a significant improvement in visual acuity at various time points, with 90% of eyes showing improvement. Intraocular pressure decreased over time. Central macular thickness increased at three months post-surgery but remained stable thereafter. Early and late complications were observed in 52.4% and 63.8% of eyes, respectively. The most common complications were posterior capsular opacification (53.3%), macular edema (26.6%), and epiretinal membrane formation (9.52%). The factors associated with complications varied between early and late stages but included age, age at the onset of uveitis, and the uveitis type. *Conclusions:* In patients with quiescent uveitis undergoing cataract surgery, significant visual improvement was achieved. This study highlights the importance of careful patient selection, preoperative and postoperative inflammation management, and precise surgical techniques. Although complications were common, the risk of capsular opacification, macular edema, and epiretinal membrane formation after surgery increased. However, future investigations should address this study’s limitations and further refine perioperative strategies.

## 1. Introduction

Uveitis is a prevalent eye disorder that results in structural and functional damage to the anterior and posterior segments of the eye due to inflammatory processes, leading to various complications [1,2]. Cataract formation is a common complication that often arises in patients with uveitis. It can affect approximately 50% to 78% of eyes with uveitis, with an annual incidence ranging from 5% to 6%, and its development depends not only on the type of uveitis but also on factors such as the duration, intensity, and effectiveness of inflammation treatment [3,4,5,6,7]. Indeed, cataract incidence in uveitis is markedly influenced by steroid treatment factors, such as the dose, duration, type, and application mode [8].

Patients with uveitis often require cataract surgery. It is suggested in the context of (1) phacoantigenic uveitis, (2) visually significant cataracts in stable eyes with a good visual prognosis, (3) hindered posterior segment examination due to cataracts, and (4) cataract extraction to enable visualization during posterior segment surgery [9]. However, in eyes with uveitis, cataract surgery presents several intraoperative and postoperative challenges, including issues like miotic pupils, synechiae, anterior segment bleeding, iris atrophy, excessive inflammation, high intraocular pressure (IOP), and cystoid macular edema (CME) [10]. These factors require careful consideration and management to achieve successful outcomes and minimize complications.

Compared with healthy eyes, cataract surgery in uveitic eyes is more complex. The complexity of surgery in uveitis necessitates consideration throughout the entire perioperative period. Before cataract surgery in uveitis patients, ensuring three months of disease inactivity is necessary and carefully selecting the type, duration, and dosage of perioperative steroids is crucial for managing inflammation and reducing complications [11,12,13,14,15]. During the surgery itself, it is often necessary to use intravitreal steroids or perform additional procedures to address structural abnormalities, such as posterior synechiae [16,17]. Effective postoperative care in uveitic eyes is of utmost importance due to consistently higher complication rates compared with eyes without uveitis and the risk of complications arising from the potential worsening or relapsing of intraocular inflammation [18,19].

Prior studies have assessed the outcomes of cataract surgery in uveitis, generally showing good postoperative visual function. The aim of this study was to evaluate preoperative conditions and postoperative early and late outcomes after cataract surgery in patients with uveitis.

## 2. Materials and Methods

This retrospective study was conducted at the University Hospital Center Rebro Zagreb, Croatia. The objective was to identify all patients diagnosed with uveitis between 2013 and 2022 who later underwent cataract surgery in the affected eye.

Patients diagnosed with uveitis who underwent cataract surgery for significant uveitic cataracts affecting visual acuity or hampering posterior segment visualization in “a quiet eye”, defined as having five or fewer cells per high-power field in the anterior chamber for at least 3 months, were included in this study.

All the patients with pre-existing retinal pathologies such as diabetic retinopathy, hypertensive retinopathy, choroidal neovascularization, any other disease that could affect the retinal thickness, or a history of previous ocular surgery or trauma were excluded from this study. Also, to minimize any potential influence on the study outcomes, we opted to exclude patients with pathologies that affected both eyes.

The data extracted from the clinical notes encompassed age, gender, age at surgery, the type of uveitis, the existence of a uveitis-associated systemic disease, pre-existing lesions, intraoperative and postoperative complications, as well as preoperative and postoperative best-corrected visual acuity (BCVA), intraocular pressure (IOP), central macular thickness (CMT), and macular volume (MV). The classification of uveitis diagnoses followed the International Uveitis Study Group’s classification of uveitis [20]. The following changes were considered postoperative complications: glaucoma, raised IOP (over 25 mmHg, which requires treatment), relapse of inflammation after surgery, posterior synechiae (seclusio or occlusio pupillae), peripheral anterior synechiae (PAS), a fibrinous reaction in the anterior chamber, iris bombe, anterior capsular fibrosis, IOL (intraocular lens) deposits, IOL intolerance, corneal edema, hypotony, posterior capsular opacification (PCO), epiretinal membrane formation (ERM), cystoid macular edema (CME), retinal detachment, and optic atrophy. All complications occurring within 6 months were categorized as early complications, while complications manifesting after 6 months were classified as late complications. Ophthalmic evaluation, including BCVA, IOP, CMT, and MV was conducted at the following time points: 1–3 days before surgery, and 1 month, 3 months, 6 months, and 12 months after surgery. For statistical analysis, the BCVA was recorded in Snellen form and then converted to a logarithm of the minimum angle of resolution (logMAR) notation. IOP was measured using the Goldman Applanation Tonometer (Haag-Streit, Köniz, Switzerland) following the application of tetracaine and fluorescein drops. The measurements were recorded in mmHg. CMT and MV were measured using spectral-domain optical coherence tomography (OCT, Spectralis; Heidelberg Engineering, Heidelberg, Germany). Values were obtained from the retinal map analysis function.

To prevent the exacerbation of uveitis following surgery, all patients received topical 1% prednisolone or 0.1% dexamethasone drops four times each day for 1 week, and an oral prophylactic corticosteroid at a dose of 0.5 mg/kg/day for 2 weeks before the surgery, and then tapered. Patients with presumed herpetic uveitis were given oral acyclovir at a dosage of 800 mg/day for 1 month before the surgery, even if they were in remission. No alterations were made to the immunosuppressive treatment protocols of the patients.

The phacoemulsification procedure was carried out by the same surgeon (M. K.). Iris retractors were used in patients with poorly dilated pupils if judged necessary. Continuous curvilinear capsulorhexis assisted with trypan blue was performed in patients with white cataracts. After completing the capsulorhexis, the lens nucleus was removed using phacoemulsification. The cortical material was removed using the irrigation/aspiration method, and then an intraocular lens (IOL) was implanted into the capsular bag. The incisions were sealed via corneal hydration, and a one-stitch corneal suture with 10–0 nylon was applied as needed. After finishing the surgery, as needed, a subconjunctival injection of 0.5 mL of dexamethasone (1 mg/mL) and 0.5 mL of gentamicin (2 mg/mL) was applied.

The patients were followed up 1 day, 1 week, 1 month, 3 months, 6 months, and 12 months after surgery. All patients receiving immunosuppressants before surgery continued their doses in the postoperative period. The postoperative medication regimen included topical 0.1% dexamethasone taken every 2 h for 3 weeks and then tapered as needed. Prednisolone was prescribed orally at 30 mg/day for the initial 3 days, followed by 20 mg/day for 2 days, and finally 10 mg/day for 2 days. In severe cases, a subconjunctival injection of 4 mg of dexamethasone was given as needed.

### Statistical Analysis

All statistical analyses were performed using IBM SPSS Statistics for Windows, version 23.0 (Armonk, NY, USA: IBM Corp.). Categorical variables were expressed as percentages and continuous variables were expressed as means ± SD. The normal distribution of the data was assessed using the Shapiro–Wilk W test. Pearson’s chi-square test was used for categoric variables to see the differences between variables, and an independent *t*-test when there were 2 independent groups or ANOVA when there were more than 2 groups was used for metric variables. To assess the differences in pre- and postoperative outcome levels, a paired-sample *t*-test was employed. Logistic regression analysis was employed to identify the risk factors associated with postoperative complications. The logistic regression analysis was performed separately for early and late complications. In each analysis, the variables that met the inclusion criteria were entered into the model to assess their independent associations with the occurrence of complications. Odds ratios (ORs) and 95% confidence intervals (CIs) were calculated to quantify the strength of associations. A *p*-value of less than 0.05 was considered statistically significant. The factors of interest included age, gender, age at onset, preoperative recurrences of uveitis, preoperative eye pathologies, and preoperative BCVA.

## 3. Results

This study comprised 105 patients (105 eyes). Five eyes of five patients were lost during the follow-up. To prevent any potential impact on the results, we excluded patients with pathologies affecting both eyes. Among these patients, 37 (35%) were male and 68 (65%) were female. The mean age ± SD was 44.33 ± 23.70, with onset at 33.57 ± 22.90 and surgery at 40.04 ± 23.84 years. The anatomical diagnoses included anterior uveitis in 67 (64%) patients, intermediate uveitis in 18 (17%) patients, and panuveitis in 20 (19%) patients. The clinical diagnoses were categorized as follows: idiopathic uveitis in 46 (44%), anterior uveitis related to HLA-B27 in 20 (19%), juvenile idiopathic arthritis (JIA) in 18 (17%), herpes simplex uveitis in 3 (3%), sarcoidosis in 10 (10%), Behçet’s disease (BD) in 1 (1%), Fuchs’ iridocyclitis (FI) in 5 (5%), and Vogt–Koyanagi–Harada disease (VKH) in 2 (2%) patients. The preoperative follow-up duration had a mean ± SD of 6.48 ± 5.94 years, and all eyes were monitored up to 12 months postoperatively. The clinical and demographic data are presented in Table 1.

The parameter scores at each time point are presented in Table 2. The mean preoperative logMAR BCVA was 1.17 ± 0.61. After surgery, there was a significant improvement in the mean logMAR BCVA observed at various time points: one month postoperatively (0.26 ± 0.39; *p* < 0.001), three months postoperatively (0.27 ± 0.36; *p* < 0.001); six months postoperatively (0.27 ± 0.34; *p* < 0.001), and twelve months postoperatively (0.23 ± 0.31; *p* < 0.001). However, it is noteworthy that the BCVA did not exhibit significant changes throughout the follow-up period (*p* > 0.05 for all time points). At the end of the follow-up period, a BCVA gain was achieved in 90% of the eyes, and 49.5% of eyes reached a BCVA of 0.2 logMAR or better. A total of 4 (3.8%) eyes remained unchanged, while 3 (2.8%) eyes exhibited a decreased BCVA. In the subgroup analysis, focusing on etiological factors with five or more eyes, the greatest visual improvement was observed in uveitis related to FI (0.04 ± 0.03), followed by JIA (0.11 ± 0.10) and idiopathic uveitis (0.26 ± 0.22), and patients with HLA-B27 uveitis demonstrated the lowest visual gain (0.35 ± 0.58) (*p* = 0.014). The linear regression analysis revealed significant associations between the postoperative visual prognosis at 12 months and preoperatively observed macular lesions (CME, EM, and PNO atrophy) (*p* = 0.040) as well as preoperative visual acuity (*p* < 0.001).

The mean ± SD intraocular pressure (IOP) before the operation was 15.24 ± 4.15. However, 12 months after surgery, it decreased to 14.22 ± 4.33 (*p* = 0.026). While there was a significant reduction in IOP at one month, three months, six months, and twelve months after surgery, the IOP did not show significant changes over the entire follow-up period (*p* > 0.05 for all time points). The lowest IOP after 12 months was observed in patients with panuveitis, with a mean of 13.70 ± 3.82.

**Figure 1:**
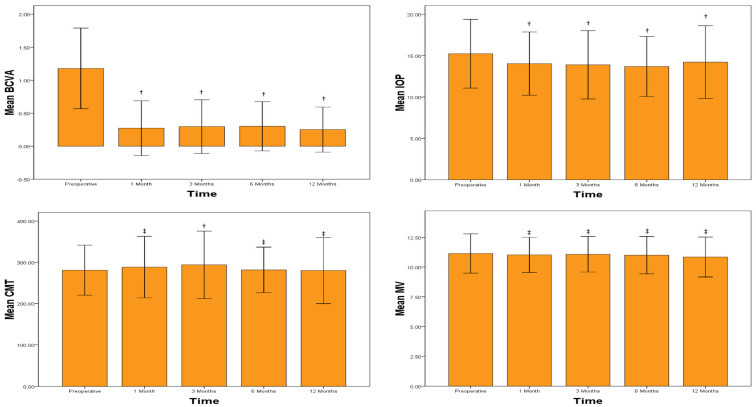
Changes in the mean values of different parameters over 12 months following cataract surgery in patients with uveitis. BCVA—best-corrected visual acuity, IOP—intraocular pressure, CMT—central macular thickness, and MV—macular volume. †—statistically significant compared with preoperative values (*p* < 0.05). ‡—no statistically significant difference compared with preoperative values (*p* > 0.05).

Three months after surgery, the central macular thickness (CMT) exhibited a significant increase compared with the preoperative measurements (*p* = 0.010). However, at the six-month mark, no significant change in macular thickness was observed (*p* = 0.890). The changes in the mean values of different parameters over 12 months are presented in Figure 1.

In the early postoperative period, complications were observed in 55 eyes (52.4%). The most commonly encountered issue was posterior capsular opacification (PCO), affecting 35 eyes (33.3%). PCO was predominantly observed in cases of idiopathic uveitis (54.2%). Among them, 24 eyes (22.8%) required Nd: YAG capsulotomy. Macular edema was observed in 18 eyes (17.1%), with predominance in cases of idiopathic uveitis (50%), followed by sarcoidosis (27.7%). One recurrence was noted in 6 eyes (5.7%) and two recurrences in 2 eyes (1.9%). One recurrence was mostly seen in idiopathic uveitis (50%), whereas two recurrences were only seen in idiopathic uveitis and JIA in 50% of cases. Late postoperative complications were observed in 67 eyes (63.8%) after cataract extraction. PCO remained the most frequent complication, occurring in 32 eyes (30.5%), necessitating Nd: YAG capsulotomy in all cases. PCO was more frequently observed in cases of idiopathic uveitis (37.5%), followed by HLA-B27-associated uveitis (25%) and JIA (21.8%). This was followed by one recurrence in 20 eyes (19%), macular edema in 16 eyes (15.2%), ERM in 10 eyes (9.5%), IOL deposits in 9 eyes (8.6%), secondary glaucoma in 8 eyes (7.6%), keratopathy in 7 eyes (6.7%), vasculitis in 6 eyes (5.7%), two recurrences in 4 eyes (3.8%), and three recurrences in 2 eyes (1.9%). One recurrence was more commonly observed in cases of idiopathic uveitis (60%), followed by HLA-B27-associated uveitis (40%). Macular edema was frequently seen in idiopathic uveitis (50%), followed by sarcoidosis (31.2%). ERM was more prevalent in cases of idiopathic uveitis (80%). Secondary glaucoma was more commonly associated with patients having JIA (50%). Keratopathy was predominantly observed in patients with JIA (28.5%) and sarcoidosis (28.5%). Newly developed macular edema was observed in 5 patients (4.7%) in early postoperative complications and in 3 patients (2.8%) in late postoperative complications. All cases were treated with a combination of oral corticosteroids and acetazolamide. Two patients received adalimumab treatment, which was administered with a loading dose of 80 mg, followed by a maintenance dose of 40 mg subcutaneously every 2 weeks. Uveitis recurrence occurred in 8 eyes (7.6%) in early postoperative complications and in 26 eyes (24.7%) in late postoperative complications. The treatment for postoperative recurrences included intensive topical corticosteroids and oral prednisolone. ERM was noted in a total of 10 eyes (9.5%) in the late postoperative period, with 5 eyes (4.7%) where it was not evident before surgery. Pars plana vitrectomy for ERM removal was necessary in 2 eyes (1.9%). Additionally, 5 eyes (4.7%) with secondary glaucoma required surgery during follow-up to manage uncontrolled increases in intraocular pressure (IOP). In our study, the most common keratopathies observed among uveitis patients who underwent cataract surgery were band keratopathy and endothelitis. These corneal complications highlight the challenges associated with phacoemulsification in patients with underlying uveitis. To gain a more detailed understanding of corneal changes and complications following cataract surgery in uveitis patients, we recognize the potential value of incorporating advanced imaging technology in future studies. Specifically, the use of anterior segment swept-source optical coherence tomography (AS-OCT) could provide invaluable insights into the structural changes and pathologies affecting the cornea after surgery [21].

In a comprehensive analysis using univariate logistic regression, we investigated various demographic and clinical factors as potential predictors of both early and late complications following cataract surgery. The factors of interest encompassed patient age, gender, age at the onset of uveitis, preoperative recurrences of uveitis, pre-existing ocular pathologies, and preoperative best-corrected visual acuity (BCVA). Our univariate logistic regression analysis yielded critical insights into the factors associated with complications. For early complications, we identified several significant predictors, including patient age, and age at the onset of uveitis. Notably, gender, preoperative recurrences of uveitis, pre-existing ocular pathologies, and preoperative BCVA did not demonstrate statistically significant associations with early complications after cataract surgery (Table 3). Turning to late complications, our analysis revealed that only preoperative BCVA was a significant predictor of these postoperative issues. In contrast, age, gender, age at uveitis onset, preoperative recurrences of uveitis, and preoperative ocular pathologies were not independently associated with the occurrence of late complications following cataract surgery (Table 4). These comprehensive findings enhance our understanding of the factors contributing to the prediction of early and late complications in uveitis patients undergoing cataract surgery. The identified predictors underscore the importance of careful patient selection, tailored preoperative and postoperative inflammation management, and precise surgical techniques in optimizing postoperative outcomes.

## 4. Discussion

Cataract development represents a significant factor contributing to visual impairment in patients with uveitis of diverse origins [22]. Achieving a successful surgical intervention is crucial for effective visual restoration and minimizing postoperative complications. Historically, cataract surgery in eyes affected by intraocular inflammation was viewed as a procedure associated with elevated risk or was even considered contraindicated due to the increased incidence of postoperative complications [23]. Consequently, phacoemulsification and IOL implantation are the prevailing methods for performing cataract surgery in uveitis patients.

The mean age of participants in our study was 44.33 ± 23.70 years, with the most common etiology being idiopathic uveitis, followed by HLA-B27 and JIA uveitis. It is noteworthy that our study’s mean age aligns with findings from previous research, although there are variations in etiological patterns between studies. For instance, Yoeruek et al. reported a mean age of 49.8 years in their study, with the predominant types of uveitis being herpes zoster iridocyclitis and sarcoidosis [8]. Estafanous et al. found a mean ± SD age of 50 ± 13 years, and the leading types of uveitis were idiopathic uveitis, sarcoidosis, and pars planitis [24]. Ram et al. reported a mean ± SD age of 42.3 ± 13.98 years, and the most frequent types of uveitis included presumed tuberculosis, VKH disease, Behçet’s disease, and sarcoidosis [25]. In a similar vein, Chang-Pin et al. reported findings consistent with our study, with a mean age of 44.6 ± 18.4 and idiopathic uveitis being the predominant etiology [26]. These variations in etiological patterns highlight the diversity in uveitis populations across different studies.

Achieving successful surgery and implementing appropriate perioperative management can lead to improved vision outcomes and facilitate a more accurate assessment of the posterior segment. In our study, over a 12-month follow-up period, visual acuity exhibited a notable improvement, transitioning from 1.15 ± 0.62 to 0.23 ± 0.31. Overall, our patients experienced significant enhancements in visual acuity, with 90% of eyes demonstrating improvement post-surgery, and approximately 50% achieving a logMAR of 0.2 or better. A total of four eyes remained unchanged, while three eyes experienced a decline in best-corrected visual acuity (BCVA). Comparatively, Kawaguchi et al. and Harada et al. reported visual improvements in 95% and 90% of eyes, respectively. In their studies, postoperative visual acuity was equal to or better than 0.3 logMAR in 87% and 82% of eyes, respectively [27,28]. Our linear regression analysis revealed that preoperatively observed macular lesions were significant risk factors associated with a poor visual prognosis (*p* = 0.040). Additionally, preoperative visual acuity (*p* < 0.001) was identified as another significant predictor of visual outcomes. Predicting the visual outcome in the eyes of patients with uveitis and a completely opacified lens can be challenging since a detailed preoperative assessment of the posterior pole is often limited. However, our study revealed that cataract extraction led to a rapid and favorable visual recovery in these patients, except for those presenting with macular lesions [29]. While our study has shown significant improvements in visual acuity for the majority of patients, it is essential to consider cases where such improvements were not observed. These cases raise important questions about the factors that may influence surgical outcomes. Specifically, it would be valuable to examine whether cases with limited visual improvement resulted in a delay in surgery for the contralateral eye.

The mean intraocular pressure (IOP) before surgery in our study was 1.15, and at the end of the follow-up period, it decreased to 14.22. This observation aligns with the findings of Rachel et al. and Deng et al., who reported a decrease in IOP from 14.9 to 13.5 and 14.32 to 13.54, respectively [29,30]. These consistent results suggest that IOP reduction can be achieved after cataract surgery in patients with uveitis.

Our study revealed that central macular thickness (CMT) did not exhibit significant changes at the end of the follow-up period. However, at the three-month mark post-operation, the CMT was found to be significantly thicker compared with preoperative values (*p* = 0.010). These findings are consistent with the research conducted by Deng et al., who also investigated CMT 12 months after cataract surgery and similarly reported no significant change in macular thickness before and after surgery [30].

While the majority of patients experienced substantial visual improvement, it is important to note that postoperative complications were relatively common. Various studies have reported different rates of complications following cataract surgery. In our study, we documented both early and late complications post-surgery. Early complications included PCO at 33.3%, and macular edema at 17.1%, with 5.7% experiencing one recurrence and 1.9% encountering two recurrences. In the case of late complications, we observed PCO at 30.5%, one recurrence at 19%, macular edema at 15.2%, ERM at 9.5%, IOL deposits at 8.6%, secondary glaucoma at 7.6%, keratopathy at 6.7%, vasculitis at 5.7%, two recurrences at 3.8%, and three recurrences at 1.9%. In comparison, other studies have reported different profiles of complications. For example, Yoeruek et al. found that the most frequent complications were postoperative uveitis recurrence (8.3%), macular edema (4.4%), PCO (38.3%), and ERM (17.2%) [8]. Estafanous et al. reported a recurrence of uveitis in 41%, macular edema in 15%, ERM in 15%, and PCO in 62% of patients in a retrospective study [24]. Meanwhile, Suresh et al. observed a prevalence of postoperative uveitis (36%) but a lower prevalence of macular edema (2%) during an average follow-up of 24.1 months [31]. The prevalence of these complications appears to be higher in studies involving extracapsular cataract extraction when compared with our study, where phacoemulsification was the surgical approach. For instance, Krishna et al. reported postoperative uveitis, macular edema, and PCO occurring in 53%, 56%, and 58% of patients, respectively, with a mean follow-up period of 81.4 months after extracapsular cataract extraction and posterior chamber intraocular lens implantation [32]. In a study by Okhravi et al., they found rates of 34%, 20%, and 32% for postoperative uveitis, macular edema, and PCO, respectively, over a shorter follow-up period (mean of 10.2 months) after extracapsular cataract extraction [5]. In comparison with our study, it is reasonable to assume that phacoemulsification has contributed to a reduction in the rate of postoperative complications. This can be explained by the fact that the smaller incision and reduced uveal trauma associated with phacoemulsification are known to decrease early postoperative inflammation and induce less damage to the blood–aqueous barrier, both of which are factors linked to the development of macular edema.

Identifying patients who may be at a heightened risk for an increased rate of postoperative complications is crucial for both preoperative counseling and postoperative care. In our study, we discovered that age and age at the onset of uveitis were factors likely to be associated with an elevated rate of early complications following cataract surgery. Interestingly, gender, preoperative recurrences of uveitis, preoperative ocular pathologies, and preoperative BCVA were not found to be predictive of early complications. For late complications, our analysis indicated that preoperative BCVA was a factor linked to a higher rate of developing complications. In contrast, age, gender, age at the onset of uveitis, preoperative recurrences of uveitis, and preoperative ocular pathologies were not identified as predictors of an increased rate of late complications after cataract surgery.

The results obtained from our study offer valuable insights that can enhance the process of patient selection and serve as a valuable tool for educating individuals with uveitis about the potential for mitigating postoperative complications following cataract surgery.

While our study has yielded valuable insights, it is crucial to acknowledge its inherent limitations. Retrospective studies, by their nature, do not allow for the complete elimination of potential bias; nevertheless, they remain instrumental in generating significant findings. The patients included in our study represented the uveitis epidemiology specific to Croatia. Notably, certain characteristics of uveitis in this population, such as the age distribution of patients and the relatively lower prevalence of posterior uveitis cases, may have exerted an influence on our results, introducing potential bias. These considerations warrant future investigation and mitigation. Nonetheless, our study has brought to light noteworthy outcomes about phacoemulsification in patients with uveitis. These findings hold promise in guiding patient counseling and facilitating the development of perioperative management strategies.

## 5. Conclusions

While performing phacoemulsification in eyes afflicted by uveitis poses a formidable challenge due to inflammation and significant structural changes, it remains a viable option for enhancing the visual prognosis of the majority of patients. This study reinforced the safety and effectiveness of phacoemulsification in uveitic eyes, underscoring the importance of meticulous patient selection, diligent preoperative and postoperative management of ocular inflammation, and a precise surgical technique. Despite the anticipated postoperative complications, the incidence of visual acuity loss remains minimal. The overwhelming majority of patients grappling with uveitis complicated by cataracts experience significant visual improvement and sustain good vision.

## Figures and Tables

**Table 1:** Summary of patient demographics (N = 105 eyes).

Characteristics	Mean ± SD or n (%)
Age (years)	44.33 ± 23.70
Age at onset (years)	33.57 ± 22.90
Age at surgery (years)	40.04 ± 23.84
Time from onset to surgery (years)	6.48 ± 5.94
Sex (male/female)	37/68 (35%/65%)
Anatomical classification	
Anterior	67 (64%)
Intermediate	18 (17%)
Panuveitis	20 (19%)
Diagnosis	
Idiopathic	46 (44%)
HLA-B27	20 (19%)
JIA	18 (17%)
Herpes simplex	3 (3%)
Behçet’s disease	1 (1%)
Sarcoidosis	10 (10%)
Fuchs’ iridocyclitis	5 (5%)
Vogt–Koyanagi–Harada disease	2 (2%)

SD—standard deviation, JIA—juvenile idiopathic arthritis.

**Table 2:** Summary of parameters over 12 months following cataract surgery in patients with uveitis.

Parameter	Time Point					*p*-Value			
	Preoperative (mean ± SD)	Month 1 (mean ± SD)	Month 3 (mean ± SD)	Month 6 (mean ± SD)	Month 12 (mean ± SD)	P vs. M1	P vs. M3	P vs. M6	P vs. M12
BCVA	1.15 ± 0.62	0.26 ± 0.39	0.27 ± 0.36	0.27 ± 0.34	0.23 ± 0.31	**<0.001**	**<0.001**	**<0.001**	**<0.001**
IOP	15.24 ± 4.15	14.04 ± 3.90	13.89 ± 4.13	13.07 ± 3.62	14.22 ± 4.33	**0.040**	**0.001**	**0.001**	**0.026**
CMT	280.99 ± 60.61	288.44 ± 74.21	293.94 ± 81.59	281.48 ± 55.68	282.01 ± 80.13	0.155	**0.010**	0.915	0.890
MV	11.18 ± 1.59	11.02 ± 1.50	11.06 ± 1.50	10.97 ± 1.55	10.92 ± 1.62	0.258	0.590	0.273	0.074

SD—standard deviation, BCVA—best-corrected visual acuity, IOP—intraocular pressure, CMT—central macular thickness, and MV—macular volume. P—preoperative, M1—Month 1, M3—Month 3, M6—Month 6, and M12—Month 12. Values are presented as means ± standard deviation. *p* < 0.05 was considered statistically significant. Factors with statistical significance are shown in bold.

**Table 3:** Factors associated with early complications following cataract surgery in patients with uveitis.

	Early Complications	
	OR (95% CI)	*p*-Value
Gender	1.10 (0.49–2.45)	0.800
Preoperative recurrences of uveitis	0.85 (0.58–1.26)	0.435
Preoperative pathologies	1.26 (0.57–2.75)	0.558
Preoperative BCVA	1.15 (0.61–2.16)	0.659
Age	1.03 (1.01–1.04)	**0.001**
Age at onset	1.03 (1.01–1.05)	**<0.001**

OR—odds ratio, CI—confidence interval, and BCVA—best-corrected visual acuity. *p* < 0.05 was considered statistically significant. Factors with statistical significance are shown in bold.

**Table 4:** Factors associated with late complications following cataract surgery in patients with uveitis.

	Late Complications	
	OR (95% CI)	*p*-Value
Age	1.01 (0.99–1.08)	0.229
Gender	0.62 (0.27–1.43)	0.269
Age at onset	1.00 (0.99–1.02)	0.363
Preoperative recurrences of uveitis	1.08 (0.73–1.59)	0.684
Preoperative pathologies	0.66 (0.29–1.48)	0.315
Preoperative BCVA	0.44 (0.22–0.89)	**0.023**

OR—odds ratio, CI—confidence interval, BCVA—best-corrected visual acuity. *p* < 0.05 was considered statistically significant. Factors with statistical significance are shown in bold.

## Data Availability

The data that support the findings of this study are available from the corresponding author, G.B., upon reasonable request.
